# Retinal Nerve Fibre Layer Thickness and Contrast Sensitivity in HIV-Infected Individuals in South Africa: A Case-Control Study

**DOI:** 10.1371/journal.pone.0073694

**Published:** 2013-09-19

**Authors:** Sophia Pathai, Stephen D. Lawn, Helen A. Weiss, Colin Cook, Linda-Gail Bekker, Clare E. Gilbert

**Affiliations:** 1 International Centre for Eye Health, Dept. of Clinical Research, Faculty of Infectious and Tropical Diseases, London School of Hygiene & Tropical Medicine, London, United Kingdom; 2 Desmond Tutu HIV Centre, Institute of Infectious Diseases and Molecular Medicine, Faculty of Health Sciences, University of Cape Town, Cape Town, South Africa; 3 Dept. of Clinical Research, Faculty of Infectious and Tropical Diseases, London School of Hygiene & Tropical Medicine, London, United Kingdom; 4 MRC Tropical Epidemiology Group, Faculty of Epidemiology and Population Health, London School of Hygiene & Tropical Medicine, London, United Kingdom; 5 Dept. of Ophthalmology, Faculty of Health Sciences, University of Cape Town, Groote Schuur Hospital, Cape Town, South Africa; Charité University Medicine Berlin, Germany

## Abstract

**Background:**

Antiretroviral treatment (ART) has altered the spectrum of HIV-related eye disease, resulting in a lower prevalence of retinal opportunistic infections (OIs). However, abnormalities in visual function have been reported in HIV-infected individuals despite effective viral suppression and the absence of retinal OIs. These changes may be mediated by an HIV-associated ‘neuroretinal disorder’, characterized by changes in the retinal nerve fibre layer (RNFL). HIV infection may also be associated with accelerated biological aging. The aim of this study was to investigate the relationships between contrast sensitivity, RNFL thickness, HIV infection and frailty in South African adults.

**Methods:**

Case-control study of 225 HIV-infected individuals without retinal OIs and 203 gender/age-matched HIV-seronegative individuals. Peri-papillary RNFL thickness was determined with spectral domain optical coherence tomography in four quadrants. CS was measured using a Pelli-Robson chart. Frailty was assessed using standard criteria. Multivariable linear and logistic regression were used to assess associations between HIV status and RNFL/CS and frailty.

**Results:**

The median age of both groups was similar (41.2 vs. 41.9 years, p = 0.37). 88% of HIV-infected individuals were receiving ART and their median CD4 count was 468 cells/μl. Adjusted CS score was lower in HIV-infected participants compared to HIV-seronegative individuals (1.76 vs. 1.82, p = 0.002). Independent predictors of poor CS in the HIV-infected group were positive frailty status and current HIV viral load >2 log copies/ml. Lower CS score was also associated with thin temporal RNFL in HIV-infected individuals (p = 0.04). Superior quadrant RNFL thickness was greatest in ART-naïve participants relative to the HIV-uninfected group (p-trend = 0.04). Longer ART duration was associated with thinning of inferior and nasal RNFL quadrants (p-trend = 0.03 and 0.04, respectively).

**Conclusions:**

Contrast sensitivity is reduced in HIV-infected individuals and functionally associated with frailty and unsuppressed viraemia. This may reflect structural changes in the RNFL that are evident despite the absence of OIs.

## Introduction

In well-resourced settings, antiretroviral treatment (ART) has altered the spectrum of HIV-related eye disease in people living with HIV infection, leading to a lower prevalence of retinal opportunistic infections (OIs) such as cytomegalovirus (CMV) retinitis [1], [2]. This trend is now becoming apparent in resource-constrained environments where scale-up of ART is commenced in individuals with increasingly high CD4 counts. However, abnormalities in visual function, such as reduced contrast sensitivity (CS), altered colour vision and visual field loss have also been reported in HIV-infected individuals despite effective viral suppression and the absence of retinal OIs. It is thought that these changes may be mediated by an HIV-associated ‘neuroretinal disorder’ (HIV-NRD) which is characterized by changes in the retinal nerve fibre layer (RNFL) [3]–[5].

One mechanism that may potentially contribute to HIV-NRD is ‘accelerated biological’ aging that is recognised to be associated with HIV infection [6]. This refers to conditions which are classically associated with the normal aging process but which occur at an earlier age in HIV-infected individuals compared with those of similar age who are seronegative [7]–[9]. This may have major implications for long-term morbidity, including for the many millions of people receiving ART in sub-Saharan Africa [10], [11]. However, there are limited data on visual function (in the absence of ocular OIs) in people living with HIV in this region. Although RNFL changes are strongly related to chronological age [12], [13], studies describing visual function, RNFL status and the possibility of HIV-NRD in HIV-infected individuals are needed, particularly as quality of life might be significantly compromised. Indeed, impaired contrast sensitivity (CS) can be more disabling than visual acuity loss [14].

We have recently undertaken a case-control study of adults in South Africa, and have shown that HIV infection is associated with several ocular changes i.e. in retinal vessel calibre, lens density and corneal endothelial cell density consistent with accelerated HIV-related aging [15]–[17]. We have also shown in the same study population that HIV infection is associated with a functional phenotype consistent with frailty [6]. This is a clinical syndrome initially described in geriatric populations, characterised by multiple pathologies, low physical activity and slow motor performance [18], [19]. Frailty predicts cognitive and physical decline and is associated with an increased risk of morbidity and mortality. We now report the findings of visual function from this study, using CS and RNFL as representative measures of ocular aging. The objectives of this study were to assess differences and identify predictors of CS and RNFL parameters in HIV-infected individuals compared to age-gender matched controls, and to assess whether visual function is related to the phenotype consistent with HIV-related accelerated biological aging that we have already reported.

## Methods

### Ethics statement

The study was approved by the Ethics Committees of the London School of Hygiene and Tropical Medicine and the University of Cape Town Faculty of Health Sciences, and adhered to the tenets of the Declaration of Helsinki. Written informed consent was obtained from all participants.

### Study participants

HIV-infected participants aged >30 years were enrolled from a community-based HIV treatment centre in Nyanga district in Cape Town [20], [21]. Participant recruitment has been reported in detail previously [6]. In brief, all participants had a confirmed serological diagnosis of HIV and were either about to commence ART (ART-naïve), or were already on first-line ART. Individuals were excluded if they had a history or current ocular OI, which was confirmed from medical case notes. All participants had a best-corrected visual acuity of 20/40 or better in order to be able to perform ophthalmic tests (i.e. CS) satisfactorily. A control group of HIV-seronegative participants was recruited from an HIV clinical prevention trials centre in a neighbouring community by frequency-matching using gender and 5-year age categories.

### Data collection

Data on age, sex, weight and height, as well as socio-behavioural factors such as housing, income, smoking and alcohol consumption were collected. Data collected for HIV-infected participants included nadir and current CD4 count, peak and current viral load, staging of HIV according to WHO clinical stage, ART status and regimen. Clinical information was obtained from medical case notes as required. Blood pressure (BP) was measured with a digital sphygmomanometer with a cuff of appropriate size. Mean arterial blood pressure (MABP) was defined as two-thirds of the diastolic plus one-third of the systolic BP [22]. Hypertension was defined as a systolic BP of 140 mmHg or higher, diastolic BP of 90 mmHg or higher, or the combination of self-reported high BP diagnosis and the use of anti-hypertensive medications [23]. Body mass index (BMI) was defined as weight (in kilograms)/height^2^. In brief physical frailty was defined by the presence of ≥3 of 5 criteria: i) unintentional weight loss (self reported and verified from clinic records where possible) ii) self-reported low physical activity, iii) self-reported exhaustion, iv) weak grip strength and v) slow walking time. Detailed assessment of frailty within this study population has been previously reported [6].

All participants underwent a full ophthalmic examination including measurement of visual acuity, evaluation by slit lamp microscopy and indirect ophthalmoscopy. Contrast sensitivity was measured with a Pelli-Robson chart under ambient (daylight) conditions as per the recommendation of the manufacturer (Haag-Streit, Essex, UK). All examinations were conducted in the same room under the same lighting conditions. The chart has eight lines of letters with two groups of three letters per line. Testing was performed at 1 m with a 0.75 diopter lens added to the manifest refraction if necessary. The logarithm to the base 10 of the CS measurement was obtained from the Pelli-Robson chart and used in analyses (LogCS). Investigators who conducted the tests were masked to other data. RFNL thickness was obtained using the Spectral OCT/SLO optical coherence tomography machine (Opko/OTI Inc, Miami, FL). OCT scans were performed by one trained operator to reduce inter-observer variation. Only OCT scans that were of sufficient quality (signal 70% of maximum strength, absence of imaging artefacts, or distortions) were used. Replicates were only taken if the OCT scans were of insufficient quality. The Spectral OCT/SLO uses a scanning laser diode of 830 nm to provide images of ocular microstructures. A peripapillary protocol inbuilt in the software was used to determine the average and quadrant-specific RNFL thickness (superior, inferior, temporal and nasal).

### Statistical analyses

One eye was randomly selected for analysis. Where an eye was not available, for example due to trauma or corneal opacity, the contralateral eye was used. LogCS scores and RNFL were initially analysed as continuous variables. Age and sex-adjusted linear regression was performed to compare logCS score and RNFL parameters respectively by HIV status. A binary categorisation of logCS was defined using the a-priori defined cutpoint of the 25^th^ percentile in the control group to denote ‘poor CS’ (1.65). Further analyses were undertaken using the cut-off of 1.50 as defined by Shah and associates [24]. Similarly, a binary categorisation of RNFL was defined using the cut-off of the 25^th^ centile in the control group to denote ‘thin RNFL’ (≤25^th^ percentile; average 101 µm, superior 118 µm, inferior 124 µm, temporal 62 µm, nasal 74 µm). Logistic regression was used to assess predictors of poor CS in those participants on ART. Multivariable linear regression models were used to examine the relationship of RNFL parameters as the dependent variable, with HIV status and other explanatory variables (age group [30–39; 40–49; >50 years], gender, MABP; BMI, smoking) as independent variables. Separate models were used for HIV-infected participants to investigate the effects of HIV-related variables. Marginal adjusted means for RNFL parameters were estimated at the mean value of covariates in the model. The Wald test was used to assess statistical significance of the association of each explanatory variable on RNFL thickness. All analyses were performed with Stata 12 (Stata Corp, College Station, TX).

## Results

### Participant characteristics

Characteristics of the 225 HIV-infected individuals and 203 HIV-seronegative age/gender frequency matched HIV-seronegative individuals are reported in Table 1. Although frequency matching was undertaken, some individuals did not have OCT images of adequate quality for analysis, resulting in a greater proportion of HIV-infected participants being included in the analyses. However, those with images did not differ from those without with respect to age, gender or other clinical/demographic characteristics (p>0.10 for all variables; data not shown). HIV-infected participants tended to have a greater income (attributable to a government health-related grant), be non-smokers, report lower consumption of alcohol and have a lower BMI than HIV-seronegative individuals. There was little difference in visual acuity between the two groups. Among the HIV-infected participants, 88% of whom were receiving ART, the current median CD4 count was 468 cells/μL (interquartile range: [IQR]: 327–607) and 84.9% had an undetectable plasma viral load (VL), defined as <50 copies/ml.

**Table 1:** Characteristics of study participants.

Variable	HIV-infected		HIV-seronegative		P-value**
	−225		−203		
Age (mean ± SE)		41.2±0.5		41.9±0.6	0.37
	N	%	N	%	
Age, years by group					
30–39	112	49.7	98	48.3	
40–49	73	32.4	65	32	0.88
>50	40	17.8	40	19.7	
Gender					
Male	60	26.7	49	24.1	
Female	165	73.3	154	75.9	0.55
Income					
<ZAR1000/month	128	56.9	140	69	0.01
>ZAR1000/month	97	43.1	63	31	
Smoking status					
Non-smoker	190	84.4	146	71.9	0.002
Smoker	35	15.6	57	28.1	
Alcohol consumption					
No	156	69.3	112	55.2	0.002
Yes	69	30.7	91	44.8	
Hypertension*					
No	144	64	122	60.1	0.41
Yes	81	36	81	39.9	
Body mass index (kg/height^2)^		28.0±0.4		31.9±0.6	0.0001
Visual acuity (presenting) (median)		20/20		20/20	0.34
		(20/20–20/25)		(20/20–20/32)	
WHO stage	N	% or median (IQR)			
2-Jan	62	27.6			
4-Mar	163	72.4			
ART naïve	27	12			
CD4 count in ART naïve group	27	170 (81–201)			
Log_10_VL in ART naïve group	16	4.79 (4.07–5.09)			
Current CD4 count in ART group		468 (327–607)			
Nadir CD4 count in ART group		136 (77–175)			
% with detectable VL in ART group	30	15.1			
Peak Log_10_VL in ART group		4.47 (3.74–4.97)			
Duration of ART, months	198	56.5 (34–74)			
ART Regimen					
Containing AZT/3TC	118	59.6			
Other	80	40.4			

* Hypertension defined as a systolic BP of 140 mmHg or higher, diastolic BP of 90 mmHg or higher, or the combination of self-reported high BP diagnosis and the use of anti-hypertensive medications.

** P-value obtained by t-test or χ^2^ –test as appropriate.

### Contrast sensitivity

Data on CS were available for 216 HIV-infected individuals and 215 HIV-seronegative individuals. Unadjusted mean logCS was lower in HIV-infected individuals compared to HIV-seronegative participants (1.77 vs. 1.82, p = 0.005) (Standard deviation and min/max values 0.22; 1.00–1.95 HIV-uninfected; 0.18; 1.20–1.95 HIV-infected). After adjustment for age, sex, smoking status (yes/no), MABP and BMI, mean logCS values still remained lower in HIV-infected individuals (1.76 vs. 1.82, p = 0.002). HIV-infected individuals were also more likely to have ‘poor CS’ (<1.65) compared to their uninfected counterparts (adjusted proportions: 43.5 vs. 31.8%, p = 0.01 – adjusted for age, gender, smoking status).

In multivariable analysis among HIV-infected participants on ART, poor CS was associated with positive frailty status (OR 3.04; 95%CI: 1.25–7.35, p = 0.01) (Table 2) and HIV viral load >2 log copies/ml (OR 3.03; 95%CI: 1.02–8.97, p = 0.05).

**Table 2:** Predictors of poor contrast sensitivity among HIV-infected participants on ART (N = 190) using logistic regression analysis.

Variable	Odds ratio (OR)	P-value
	For reduced contrast sensitivity	(from likelihood ratio test; LRT)
Age group		
30 years	1	
per 10 year increase	1.22 (0.75–2.00)	0.43
Sex		
Male	1	
Female	1.73 (0.66–4.57)	0.27
Frailty status		
Not frail	1	
Frail	3.04 (1.25–7.35)	0.01
Smoking status		
Non-smoker	1	
Current	0.49 (0.17–1.45)	0.2
Current CD4 count		
<400 cells/μl	1	
>400 cells/μl	0.84 (0.40–1.77)	0.64
Nadir CD4 count		
<100 cells/ μl	1	
100–200 cells/ μl	1.66 (0.79–3.50)	0.28
>201 cells/μl	2.30 (0.69–7.63)	
Current viral load		
<2 log copies/ml	1	
>2 log copies/ml	3.03 (1.02–8.97)	0.05
Peak viral load		
<4.5 log copies/ml	1	
>4.5 log copies/ml	0.85 (0.43–1.68)	0.64
WHO clinical stage		
2-Jan	1	
4-Mar	1.84 (0.78–4.32)	0.17
ART duration (months)		
<24	1	
25–48	2.28 (0.74–7.08)	
49–72	2.73 (0.85–8.75)	0.3
>73	1.68 (0.50–5.61)	
ART regimen		
Containing AZT/3TC	1	
Other	1.20 (0.61–2.34)	0.6

* adjusted for all variables in table, and mean arterial blood pressure and BMI, age group used as a linear term in model.

### Retinal nerve fibre layer

Table 3 shows the average and quadrant RNFL thickness stratified by HIV status and by viral load status. Average RNFL thickness was similar between two groups as stratified by HIV status (yes; no) (109.7±0.8 μm in HIV-infected participants, vs. 108.7±0.9 μm in HIV-seronegative individuals, p = 0.41) There was a trend of greater RNFL thickness of the superior quadrant with greater HIV viremia: the mean superior RNFL thickness in HIV-seronegative individuals was 132.2 μm, compared to 133.8 μm in HIV-infected individuals on ART with non-detectable VL, and 140.0 μm in HIV-infected individuals who were ART-naïve (and had detectable VL), p-trend = 0.04. A similar trend was observed for the inferior quadrant (p-trend = 0.13). Associations of RNFL thickness with current or nadir CD4 count were not observed.

**Table 3:** RNFL thickness in μm (standard error) by HIV and viral load status – adjusted for age, gender, mean arterial blood pressure, smoking status and BMI.

RNFL	HIV−	All HIV+	P-value^a^	On ART	On ART	ART-naive	P-value^b^
Thickness in μm				VL<50 copies/ml	VL>50 copies/ml		(Wald test)
(SE)				(n = 168)	(n = 30)	(n = 27)	
	(n = 203)	(n = 225)					
Average**	108.7	109.7	0.41	109	111.9	111.3	0.52
	−0.9	−0.8		−1	−2.4	−2.5	
Superior	132.2	135.1	0.16	133.8	138.5	140	0.04*
	−1.5	−1.4		−1.6	−3.8	−4.1	
Inferior	137.9	135.6	0.27	134.7	137.7	139.5	0.5
	−1.5	−1.4		−1.7	−4	−4.3	
Nasal	88.4	91.1	0.19	91.2	89.5	95.1	0.13*
	−1.5	−1.5		−1.7	−4	−4.4	
Temporal	72.5	73.1	0.65	73.1	73.3	70.7	0.85
	−0.9	−0.9		−1	−2.4	−2.7	

Multivariable linear regression model; marginal adjusted means for RNFL parameters were estimated at the mean value of covariates in the model.

** Limited images in this group; total n = 402 (188/160/28/26).

a – for difference between HIV-infected and HIV-seronegative groups.

b – for difference between HIV-seronegative and HIV groups by viral load status.

* – refers to p-value using test for trend.

**Figure 1:**
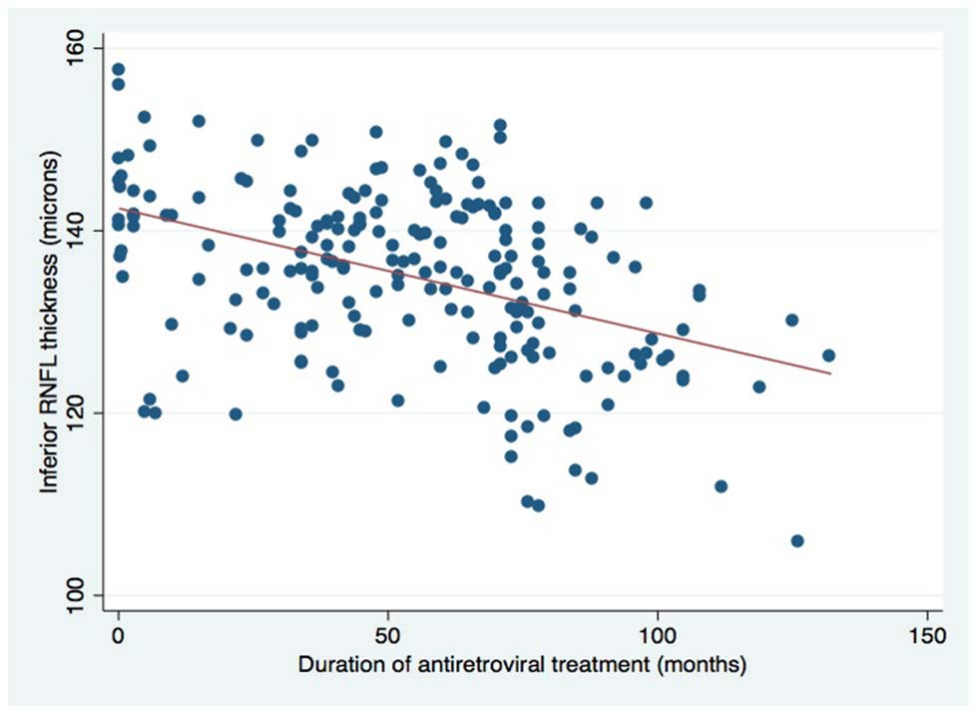
Scatterplot showing inferior retinal nerve fibre thickness (RNFL) vs. antiretroviral treatment duration. Refer to Table 4 for further information.

**Figure 2:**
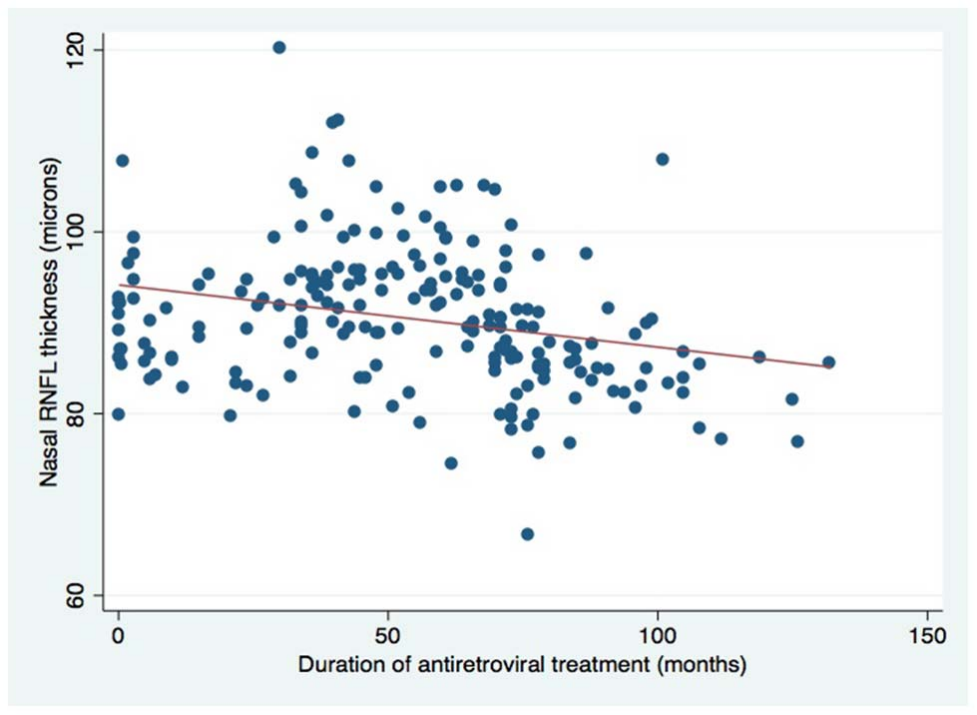
Scatterplot showing nasal RNFL vs. antiretroviral treatment duration. Refer to Table 4 for further information.

Among HIV-infected participants increasing age was, as expected, associated with thinning of the RNFL. Increasing duration of ART was associated with reduced thickness of the RNFL in inferior and nasal quadrants (Table 4 and Figures 1 and 2). No association was detected with type of ART. Average and superior quadrant RNFL thickness was lower in those with advanced HIV infection at ART initiation, as defined by WHO clinical stage 3 or 4.

**Table 4:** RNFL thickness in μm in HIV-infected participants (n = 188).

		Retinal nerve fibre layer thickness (μm)									
Parameter	N	Average		Superior		Inferior		Nasal		Temporal	
		μm p-value		μm p-value		μm p-value		μm p-value		μm p-value	
Age group, years											
30–39	95	112		138.3		139.7		89.3		76.6	
40–49	61	109.5	<0.0001*	133.7	0.004*	133.4	0.001*	93.7	0.29	70.6	0.006*
>50	32	102.2		124.9		125.2		88.5		69.3	
ART duration, months											
<36	55	110.4		136.6		138.5		93		72.3	
37–72	83	110.1		135.1		137		93.4		73.2	
>73	50	107.7	0.37*	131.4	0.27*	128.6	0.02*	84.1	0.04*	74.5	0.81
WHO clinical stage											
2-Jan	42	112.5		141.1		139.7		90.2		74.7	
4-Mar	146	108.7	0.07	132.7	0.03	133.8	0.14	90.7	0.92	72.9	0.56
Current viral load											
<2 log copies/ml	163	109.2		134.1		134.8		91.1		73	
>2 log copies/ml	25	111.8	0.37	137.1	0.58	136.5	0.92	87.5	0.29	75.4	0.47

* = p-value for test of trend; otherwise P-value derived from Wald test.

Multivariable linear regression model; marginal adjusted means for RNFL parameters were estimated at the mean value of covariates in the model.

Adjusted for age, gender, smoking status, mean arterial blood pressure, BMI, nadir and current CD4 count, ART regimen and all parameters displayed in table.

### Relationship between CS and RNFL thickness

**Figure 3:**
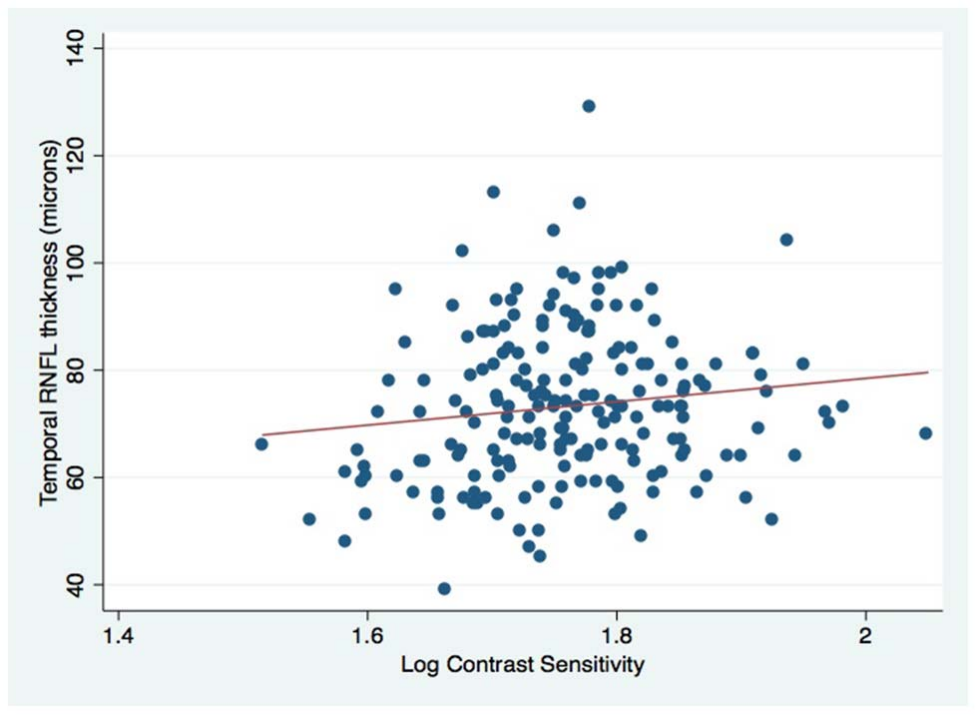
Scatterplot showing temporal RNFL thickness vs. log contrast sensitivity values.

Finally, we assessed CS score according to RNFL thickness (thin or normal) in HIV-infected participants. Lower CS was associated with thin temporal RNFL (1.70; thin RNFL, vs. 1.78; normal RNFL, p = 0.04); the association of temporal RNFL thickness with CS is also depicted in Figure 3. Logistic regression to assess the predictive value of temporal thinning of the RNFL as a risk factor for poor CS showed that those with thin temporal RNFL had a three-fold risk of poor CS (≤1.5), OR: 3.39 (95%CI: 1.22–9.44, p = 0.02).

## Discussion

This study provides evidence that HIV infection is strongly associated with poor CS in this South African population. Data from other regions, particularly the USA, demonstrate evidence of an HIV-related ‘neuro-retinal disorder’, comprising subtle vision abnormalities in the absence of opportunistic infections [3]–[5], [24]. However, this has not been investigated in sub-Saharan Africa to date, where access to ART and the aging HIV-infected population both continue to increase. In a study population from this region we assessed both structural and functional components of the RNFL. Our findings relating to RNFL thickness are novel: higher levels of HIV viremia were associated with a thicker RNFL, whereas longer duration of ART was associated with a thinner RNFL. We also found frailty to be an important predictor of poor CS. These findings have potentially important implications for long-term visual function particularly among the expanding HIV-infected aging population in sub-Saharan Africa.

We have previously demonstrated that HIV infection is associated with an increased risk of frailty, providing evidence that this functional phenotype is associated with HIV-related accelerated aging [6]. The present study builds on these findings, demonstrating that frailty is an important predictor of poor CS. This suggests that there may be a visual component to frailty in HIV infection. This is further corroborated by the finding that abnormal CS is also independently associated with mortality in individuals with AIDS [25], and may be a sensitive indicator of generalized aging [26]. However, the present criteria for frailty assessment do not contain any visual function indicators. In light of these findings, a validation study that includes visual function as another measure of frailty may be warranted. Changes in the RNFL have been demonstrated in conditions with neuro-cognitive decline (e.g. Parkinson's Disease; Alzheimer's Disease) [27], [28] and a neuro-cognitive component may also contribute to the frailty phenotype [29], [30], as well as HIV-associated neurocognitive disorders [31]. Therefore measurement of RNFL may also be a useful novel predictor in this context.

Although the frequency of poor CS was markedly higher in HIV-infected individuals compared to controls, the median visual acuity between the two groups was the same and all participants had visual acuity better than 20/40. Contrast sensitivity loss can be present even when visual acuity and fields are relatively intact [32]. It is also a better predictor of mobility performance than visual acuity [33]. These findings highlight that visual complaints from HIV-infected individuals may be related to poor CS, and that visual acuity testing (often performed as a baseline ‘screen’ by HIV physicians to guide further referral) will often be normal. Assessment of CS could be a useful aid in initial examination of patients with symptoms suggestive of visual dysfunction.

In the context of accelerated aging and HIV, reduced thickness of the RNFL in HIV-infected individuals compared to uninfected counterparts of similar age might be expected as the RNFL thins with increasing chronological age [12], [13]. Other studies have compared RNFL thickness in HIV-infected individuals with HIV-seronegative controls, but it is difficult to make comparisons as study populations differ in terms of demographic and HIV-related characteristics as well as in the methods used to assess RNFL. Thinning of the RNFL has been detected in HIV-infected participants in the USA with low nadir CD4 count when compared with HIV-infected individuals with higher nadir CD4 count and HIV-seronegative controls in using OCT [4], [34]. Significant thinning of the RNFL in HIV-infected participants (regardless of CD4 count) compared to uninfected controls, was observed with confocal scanning laser tomography [35] whereas a study from Brazil did not find a significant difference in RNFL thickness between HIV- infected and uninfected participants using OCT [36]. In our study, we did not observe an overall difference in RNFL thickness between the two groups, nor we did we detect associations related to CD4 count and RNFL thickness, although we did detect mild associations with HIV viremia. It is therefore difficult to place our study in the context of previous work, as the epidemiology of HIV in sub-Saharan Africa is likely to be different to that in other regions. In addition, the HIV and ART ‘trajectory’ may be at earlier stages compared to HIV cohorts in well-resourced settings (where the majority of studies have been conducted) thus, our findings may reflect those seen relatively early on in chronic HIV infection.

Our findings suggest that ART duration is an important factor in determining RNFL thickness (inferior and nasal quadrants) after adjusting for age and other co-variates, and we have previously shown that narrower retinal arterioles are associated with increasing duration of ART, independently of age [15]. RNFL thinning and retinal arteriolar narrowing may be related to early vascular dysfunction in the nerve fibre layer, mediated by either HIV infection or ART. This is further substantiated by our finding that RNFL thinning was observed mainly in the superior and inferior quadrants, where the majority of large retinal vessels are located. Longitudinal studies are required to evaluate the contribution of ART and HIV infection to possible accelerated aging changes in the RNFL.

The association of detectable HIV viremia with an *increase* in RNFL thickness in our study population may be biologically plausible. Medzhitov [37] used the term “para-inflammation” to describe an intermediate tissue adaptive response: infection and injury lead to full inflammation, whereas chronic tissue stress initiates mild low-grade ‘para’-inflammation, a mechanism which attempts to maintain tissue homeostasis and monitor tissue malfunction. However, chronic para-inflammation can lead to disease progression and is thought to be an important process in age-related retinal diseases [38]. Increased levels of HIV viremia might initiate a para-inflammatory process in the retina, which may manifest as increased thickness of the RNFL. Kalyani and associates [3] have also reported greater RNFL thickness in a subgroup of HIV-infected individuals in the USA. They suggested that mitochondrial toxicity (mediated by HIV or ART) may cause axonal damage to the RNFL, leading to an initial phase of swelling before atrophy. Similar mechanisms are postulated in Leber's hereditary optic neuropathy (LHON), a mitochondrial disorder where mitochondrial dysfunction leads to degeneration of retinal ganglion cells and their axons in the optic nerve [39]. OCT measurements in LHON patients show that RNFL thickness increases in the pre-symptomatic stage, followed by a reduction over time [40]. Additionally, we found that lower CS score was associated with thinner RNFL in the temporal quadrant, a finding also demonstrated by Kalyani and associates [3]. Anatomically, this could reflect preferential damage to the small-calibre axons of the maculopapillary bundle, similar to proposed mechanisms in LHON [3], [39].

A key strength of this study is the inclusion of an age/gender-matched control group with a similar socio-demographic profile as the HIV-infected individuals. By recruiting from the same community, we aimed to reduce the likelihood of differential risk exposure in line with the recommendation for careful study design when investigating premature aging in HIV [41]. Moreover, this allowed us to directly reference findings in HIV-infected individuals to a comparable control population, rather than recourse to normative data from other populations. As there are few data relating to normative visual function or RNFL in African populations, reference to other ethnic or geographical populations could produce erroneous comparisons. Our study population is also large compared to several studies of RNFL and visual function in HIV, and we used the same OCT machine and operator for the duration of the study to minimize potential variability in measurements, which can occur with different OCT machines and operators [42].

This study had some limitations. The study design means we cannot infer whether HIV is causally related to poor CS function or changes in RNFL, nor can a temporal relationship be established. The measurement of CS is subjective, however we adhered to standardized protocols (e.g. lighting, measurement distance) and used the same chart and examiner in the same location. Another potential limitation is that misclassification of smoking and alcohol consumption may have occurred, with HIV-infected participants wanting to demonstrate ‘healthy behaviour’ which could have led to confounding. Finally, in the context of further defining the HIV-neuroretinal disorder it would have been ideal to have data on visual fields and colour vision, however, this initial data provide an informative basis from which to plan further studies in this study population or region. Further investigations such as micro-perimetry and retinal electrophysiology would also help to elucidate possible mechanisms and pathways of HIV-associated neuro-retinal disorder.

In summary, HIV-infected individuals in South Africa demonstrate abnormal CS, and changes in RNFL thickness related to viremic status. Longitudinal studies are needed to determine whether changes in RNFL and systemic indicators such as frailty predict change in visual function, and equally importantly, the role of ophthalmic indicators in predicting the biologically aged phenotype in chronic HIV infection.
